# The discovery and importance of genomic imprinting

**DOI:** 10.7554/eLife.42368

**Published:** 2018-10-22

**Authors:** Anne C Ferguson-Smith, Deborah Bourc'his

**Affiliations:** 1 Department of GeneticsUniversity of CambridgeCambridgeUnited Kingdom; 2 Genetics and Developmental Biology DepartmentInstitut CurieParisFrance

**Keywords:** genomic imprinting, epigenetics, DNA methylation, germline, mammalian reproduction, epigenetic inheritance

## Abstract

The discovery of genomic imprinting by Davor Solter, Azim Surani and co-workers in the mid-1980s has provided a foundation for the study of epigenetic inheritance and the epigenetic control of gene activity and repression, especially during development. It also has shed light on a range of diseases, including both rare genetic disorders and common diseases. This article is being published to celebrate Solter and Surani receiving a 2018 Canada Gairdner International Award "for the discovery of mammalian genomic imprinting that causes parent-of-origin specific gene expression and its consequences for development and disease".

## Imprinted genes in development, epigenetics and disease

In 1984, Davor Solter (working with James McGrath at the Wistar Institute in Philadelphia) and, independently, Azim Surani (working with Sheila Barton and Michael Norris at the AFRC Institute of Animal Physiology in Cambridge) published the results of experiments on newly fertilized mouse eggs (McGrath and Solter, 1984; Surani et al., 1984; Barton et al., 1984). They had generated embryos that contained either two sets of chromosomes inherited from the mother, or two sets of chromosomes inherited from the father. However, when transferred into pseudo-pregnant recipient females, the embryos failed to develop to term.

These remarkable results sent a clear message: despite being genetically equivalent, the set of chromosomes inherited from the mother were not functionally equivalent to the set inherited from the father. The defective development of the bi-maternal and the bi-paternal embryos indicated that, for normal development to occur, one set of chromosomes from each parent was required. This is due to a process called 'genomic imprinting' which acts in the gametes to 'mark' genes on the maternal and paternal chromosomes in order to ensure parent-of-origin specific expression after fertilization. All cells contain two copies of every gene (except those genes found on the single Y chromosome in males). In general both copies of a gene are expressed. However, cells express only one copy of an imprinted gene – either the copy inherited from the father or the copy inherited from the mother. It later emerged that the imprinting marks were epigenetic modifications (in particular, DNA methylation).

Around the same time, genetic studies by Bruce Cattanach and Michael Kirk showed that imprinted genes were not evenly distributed across the whole genome but located in particular genomic regions (Cattanach and Kirk, 1985). This finding was confirmed by the subsequent identification and mapping of imprinted genes, although the first three imprinted genes, *Igf2r, Igf2 and H19*, were not identified until 1991 (Barlow et al., 1991; DeChiara et al., 1991; Ferguson-Smith et al., 1991; Bartolomei et al., 1991).

Over the years, studies in mice and humans have shown that imprinted genes are essential not only for the prenatal development of normal embryonic and extraembryonic components, as demonstrated in the early experiments of Surani and Solter, but also for postnatal processes that include the regulation of the brain and behavior, metabolism, and physiological adaptations (Cleaton et al., 2014). Moreover, a number of human syndromes exhibiting parent-of-origin effects in their patterns of inheritance were known, including the fetal overgrowth disorder Beckwith-Wiedemann Syndrome (which is also associated with an increased incidence of childhood tumors), and two neurological disorders (Prader-Willi Syndrome and Angelman Syndrome). These and other syndromes were found to be caused by the inheritance of two imprinted domains from the mother and none from the father, or vice versa; by deletions at imprinted regions; or by a failure either to establish a proper imprint during gamete production or to maintain it after fertilization.

Such studies have, of course, been important for elucidating these imprinted disorders, but perhaps more importantly, they have implicated imprinted genes more generally in pathways that control the aetiology of much more common diseases, such as those involved in growth, metabolism, cancer and neurological disorders.

We now know that genomic imprinting involves the transmission of epigenetic information, in the form of DNA methylation marks, from gametes to offspring, with the result that a set of around 100–200 genes (both protein coding genes and non-coding RNA genes) are expressed from only one of the two chromosomes in cells. The essential role for DNA methylation in imprinting was shown through the inheritance of mutations in DNA methyltransferases (Bourc'his et al., 2001; Li et al., 1993; Kaneda et al., 2004). These DNA methylation marks provide an imprint that is acted upon by a hierarchy of transcriptional and chromatin states, including differential histone modifications on the two parental chromosomes (Fournier et al., 2002), that result in the monoallelic expression of imprinted genes.

It also became clear that imprinted genes are often clustered around a single imprinting control region (ICR) that influences the monoallelic expression of the whole cluster. Indeed, ICRs are regulatory sequences that control the expression of genes that code for proteins, or for long-non-coding RNAs that control the activity of the cluster in cis. A transcription factor called CTCF also has an important role at some (but not all) of these clusters, to modulate the regulation of imprinted gene expression in a parental-origin-specific manner.

Hence, over the years, the analysis of differential DNA methylation at imprinted domains has provided a paradigm in which to assess the links between particular epigenetic states and the long- and short-range cis-acting control of gene expression in mammals. These studies have uncovered regulatory relationships between DNA methylation, histone modifications, long-non-coding RNAs and associated proteins (such as CTCF), and has helped to define many of the enzymatic processes that write, read and erase epigenetic states (Barlow and Bartolomei, 2014; Ferguson-Smith, 2011).

## Imprinted genes: a paradigm of epigenetic regulation with genetic determinism

Where do we stand now, 34 years after the original discovery of genomic imprinting? High throughput sequencing approaches – and their application to small numbers of cells – have been instrumental in revealing the developmental regulation and extent of genomic imprinting.

In particular, genome-wide profiling of gametic methylation in mice and humans has highlighted that thousands of sequences acquire asymmetric DNA methylation states in the oocyte and spermatozoon, reflecting the contrasting biology of DNA methylation in the two germlines (Smallwood et al., 2011; Kobayashi et al., 2012). In males, sperm methylation preferentially targets intergenic sequences and transposon repeats. In females, oocyte methylation coincides with the body of actively transcribed genes, including intragenic CpG islands (Veselovska et al., 2015). The distribution and genomic properties of ICRs do not differ from these genome-wide trends: maternal ICRs all coincide with CpG island promoters located downstream of transcription start sites that are active during oocyte growth, while paternal ICRs have an intergenic location. ICRs are not established as special regulators in the germline: rather they are selected, post-fertilization, by being actively protected from the genome-wide loss of methylation that occurs before embryo implantation.

The realization that the epigenetic protection of ICRs was genetically determined came as a surprise. ICRs are endowed with several TGCCGC motifs and, when methylated, these motifs are recognized by a zinc finger protein called ZFP57 which, in turn, recruits the KAP1-centered heterochromatic complex (Quenneville et al., 2011). This allows for the concentration of DNA methyltransferases around the ICRs and the propagation of their germline-methylation status in the early embryo, while the rest of the genome is undergoing global reprogramming. Upon complete depletion of ZFP57, multiple ICRs fail to maintain their parental methylation imprint after fertilization, leading to misregulation of imprinted expression and embryonic lethality (Li et al., 2008). More generally, all cases of epigenetic intergenerational inheritance – both normal and pathological – may follow the same molecular principle: to persist in the next generation, genomic sequences that have acquired methylation during gametogenesis have to be recognized by methyl-sensitive, sequence-specific DNA binding factors (such as ZFP57) to locally attract the DNA methylation enzymes.

Though we still do not understand the evolutionary processes that have led to the emergence of genomic imprinting in mammals, its interrogation over the years has revealed a wealth of epigenetic insight that continues to have an enduring influence on genome biology.

Incidentally, CpG-rich sequences, such as CpG islands, have a higher probability of containing several TGCCGC motifs and, therefore, a higher probability of being protected by ZFP57. This may explain the greater number of maternal ICRs (which are CpG-rich promoters) compared to paternal ICRs, which are derived from intergenic sequences and are, therefore, under lower evolutionary pressure to maintain CpG motifs. The current census is around 22 maternal ICRs versus three paternal ICRs, hence a total of 25 ICRs. Paternal ICRs are not only at a numerical disadvantage, they also have less influence on development than maternal ICRs (Schulz et al., 2010). Nonetheless, it is noteworthy that among the diverse efforts that followed the original work of Solter and Surani to modify parental imprinting and overcome the barriers to mono-parental reproduction, viable bi-maternal mice were produced (Kawahara et al., 2007). This was done by aggregating a normal maternally-imprinted genome with a second non-imprinted maternal genome in which the imprinting effects of two of the three paternal ICRs were restored through the use of genetic deletion.

## Variations of imprinting in space and time

Many genome-wide screens have been developed to identify new imprinted loci but the general conclusion is that all the canonical ICRs – that is, those with parent-specific DNA methylation patterns that are maintained in a life-long and tissue-wide manner – have probably been uncovered (Xie et al., 2012). Less robust ICRs have been detected, whereby parent-specific DNA methylation patterns are confined to early embryonic development or persist in some adult tissues only (Proudhon et al., 2012). These stage- and tissue-specific ICRs translate into variations in the allelic dosage of the imprinted genes they regulate (Greenberg et al., 2017). Biallelic expression of imprinted genes can also occur without modifying the methylation imprint itself: for example, the paternally expressed *Dlk1* gene adopts biallelic expression in neural stem cells with important implications for neurogenesis (Ferrón et al., 2011). These studies have highlighted that genomic imprinting is more flexible across the lifetime of an individual than originally thought. But imprinting can also be polymorphic between mammalian species: some ICRs are found in rodents but not in humans, and *vice versa* (see, for example, https://atlas.genetics.kcl.ac.uk/atlas.php and www.geneimprint.com/site/genes-by-species).

As for many genetic innovations, transposons have acted as major drivers for the emergence of these species-specific ICRs. During spermatogenesis, retrotransposon methylation is guided by specific small RNAs called piRNAs (short for PIWI-interacting RNAs) to ensure that the germline genome is protected. Through this mechanism, a retrotransposon that landed into the *Rasgrf1* locus in rodents has created a new paternally methylated ICR in these species (Watanabe et al., 2011). Similarly, long terminal repeat (LTR) sequences of specific retrotransposons are particularly active during oogenesis. Through their promoter activity, they can define new transcription start sites in oocytes and promote transcription-coupled DNA methylation of downstream CpG islands (Brind'Amour et al., 2018). Rodent- and human-specific insertions that contain binding sites for post-fertilization methylation maintenance (by factors such as ZFP57) can, therefore, diversify the germline 'methylome' and generate new species-specific ICRs. Furthermore, because LTR transposons are still active in rodent genomes, some ICRs are found in some mouse strains but not in others (Brind'Amour et al., 2018). Whether these could be phenotypically influential is unknown.

## Non-canonical genomic imprinting

Finally, one of the most intriguing findings of recent years was the discovery of genomic imprinting that does not involve DNA methylation. The trimethylation of lysine 27 in histone H3 (H3K27me3) is an epigenetic mark that is asymmetrically transmitted by parental gametes and remains after fertilization to influence the allelic expression of several genes in the early embryo (Inoue et al., 2017). However, this 'non-canonical' form of genomic imprinting is exclusively transmitted by the oocyte and is only maintained until the blastocyst stage. By the time of implantation – less than a week after fertilization – the parent-specific differences have disappeared.

Genomic imprinting was discovered at a time when the modifications to DNA and chromatin that act 'on top of' genetics and regulate genome function were only beginning to be appreciated. The contribution of this essential mammalian developmental process to our understanding of epigenetic mechanisms has been major. Through the analysis of active and repressed alleles of imprinted genes within a given cell type during development, robust relationships between regional epigenetic control and transcriptional behavior have been established. Though we still do not understand the evolutionary processes that have led to the emergence of genomic imprinting in mammals, its interrogation over the years has revealed a wealth of epigenetic insight that continues to have an enduring influence on genome biology.
